# Baicalin reverses radioresistance in nasopharyngeal carcinoma by downregulating autophagy

**DOI:** 10.1186/s12935-020-1107-4

**Published:** 2020-01-30

**Authors:** Cong Wang, Yinli Yang, Lining Sun, Jing Wang, Zhansheng Jiang, Yanwei Li, Dongying Liu, Haiyan Sun, Zhanyu Pan

**Affiliations:** 1 Department of Integrated Traditional & Western Medicine, Tianjin Medical University Cancer Institute and Hospital, National Clinical Research Center for Cancer, Key Laboratory of Cancer Prevention and Therapy, Tianjin’s Clinical Research Center for Cancer, Huan-Hu-Xi Road, Ti-Yuan-Bei, He Xi District, Tianjin, 300060 China; 2 Department of Radiation Oncology, Fudan University Shanghai Cancer Center, Shanghai, 200032 China; 3 Department of Radiation Oncology, Tianjin Medical University Cancer Institute and Hospital, National Clinical Research Center for Cancer, Key Laboratory of Cancer Prevention and Therapy, Tianjin’s Clinical Research Center for Cancer, Tianjin, 300060 China

**Keywords:** Baicalin, Radioresistance, Nasopharyngeal carcinoma, Autophagy

## Abstract

**Background:**

Radiation resistance is the main cause of recurrence after radiotherapy, and increased autophagy after radiotherapy is related to radiotherapy resistance. This study aims to investigate the reversal effect of baicalin on radioresistance and its related mechanism.

**Methods:**

CCK-8 and flow cytometry were used to detect the effect of proliferation and apoptosis by baicalin. Clone formation test was used to verify the effect of baicalin radiosensitization. Western blot analysis and electron microscopy were employed to observe the effect of baicalin on autophagy.

**Results:**

Compared with the radiation therapy (RT) group, the RT combined baicalin (RT + BA) group showed a significantly low 2 Gy survival fraction of radiation therapy (P < 0.05). LC3-II protein expression in the RT group was significantly higher than which in the RT + BA group (P < 0.05). Electron microscopy showed that more autophagic vacuoles were observed in the RT group than those in the RT + BA group.

**Conclusions:**

Overall, baicalin can reverse the radioresistance of human nasopharyngeal carcinoma CNE-2R cells by downregulating RT-enhanced autophagy.

## Background

Approximately 80,000 new cases of nasopharyngeal cancer are reported worldwide yearly, accounting for 50% of all head and neck malignant cancers [1]. Radiation therapy is the main treatment for nasopharyngeal cancer due to late staging, local anatomical limitations, invasive growth characteristics, and sensitivity to radiation [2]. Nonetheless, approximately 30–40% of patients receiving radiation therapy may have residual or recurrent lesions. Resistance to radiation therapy is the main cause of recurrence after this type of treatment. The five-year survival rate of patients with recurrence is only 25–35%, and the prognosis is also extremely poor [3].

Autophagy is a conservative and highly close pathway to intracellular clearance and circulation, which maintains cell metabolism and survival during periods of starvation or stress. Autophagy is also closely related to tumor occurrence and development [4]. Radiotherapy can induce autophagy of cancer cells through multiple pathways [5]. According to the study of radioresistant human nasopharyngeal carcinoma cell line CNE-2R, radiotherapy induces autophagy of nasopharyngeal carcinoma cells, and inhibition of autophagy can reverse radioresistance [6].

*Scutellaria baicalensis*, a traditional Chinese medicine, is derived from the dry root of *S. baicalensis* and has a wide range of clinical applications. The pharmacological effects of *S. baicalensis* include antipyretic, analgesic, anti-inflammatory, anti-bacterial, anti-tumor, anti-virus, scavenging oxygen free radicals, and antioxidant [7, 8]. Baicalin is the most abundant component of *S. baicalensis*. Baicalin reduces growth of nasopharyngeal carcinoma in vivo and in vitro, and induces cell cycle arrest and apoptosis [9]. In this study, molecular and cell biology techniques were used to investigate whether baicalin reverses the radioresistance of nasopharyngeal carcinoma through autophagy inhibition.

## Materials and methods

### Reagents and cell culture

Human nasopharyngeal carcinoma cell line, CNE-2, was obtained from the Fudan University Shanghai Cancer Center (Shanghai, China) and maintained at 37 °C with 5% CO_2_ in RPMI 1640 medium (Gibco, Thermo Fisher Scientific, Waltham, MA, USA) supplemented with 10% fetal bovine serum (Gibco, Thermo Fisher Scientific, Waltham, MA, USA). CNE-2R, a radiation-resistant human nasopharyngeal carcinoma cell line, was constructed and cultured in the Immunology Department of Tianjin Medical University Cancer Institute and Hospital. The purity of baicalin (Sigma, St. Louis, MO, USA) was larger than 95%. Baicalin was diluted in sterile dimethyl sulfoxide (DMSO, Sigma) and stored in the dark at 4 °C.

### Irradiation procedure

Cell line irradiation was performed by a 6 MV X-ray beam from the Varian Trilogy Linac (Varian Inc., USA) at a dose rate of 220 cGy/min.

### Clonogenic survival assays

CNE-2R cells were seeded in the six-well plates at concentrations of 200, 400, 800, 1000, and 2000 cells/well. The cells were pretreated with or without 10 μg/ml baicalin for 24 h and then exposed to 0, 2, 4, 6, or 8 Gy X-ray beam dose. The medium was then replaced with drug-free RPMI 1640, and the cells were cultured for 10–14 days with 5% CO_2_. Colonies were then stained with 0.5% crystal violet (Sigma, St. Louis, MO, USA) solution for 10 min and counted using light microscopy at 40× magnification. A viable colony was defined as having at least 50 cells. Dose–response curves were analyzed using the multitarget single-hit model in the GraphPad Prism 5.0 software.

### Cell proliferation assays

Proliferation was measured using CCK8 assay. CNE-2R cells were seeded into 96-well plates (3 × 10^3^ cells/well) and pretreated with various concentrations of baicalin for 24 h. CCK8 (10 μg/ml) was added into the plates, and then the absorbances at 450 nm after 3 h were recorded (Biotek Instruments Inc., Winoski, VT, USA). Five replicate wells were evaluated in each group, and three independent experiments were performed. Cell survival was calculated using the following formula:

$$ {\text{Survival rate }}\left( {\text{\%}} \right) = \frac{OD}{{OD_{0h} }} \times 100{\text{\% }} $$

### Apoptosis assay

FITC-conjugated annexin V was used to detect apoptosis. CNE-2R cells were seeded in 24-well plates (1 × 10^4^ cells/well) for 24 h before radiation exposure (4 Gy). After irradiation, the cells were incubated for 24 h and then harvested and stained using the annexin V-FITC/PI apoptosis detection kit (Invitrogen, Inc., Carlsbad, CA, USA) according to the manufacturer’s instructions. The resulting fluorescence was detected using flow cytometry.

### Western blot analysis

CNE-2R cells were seeded overnight in a six-well plate (6 × 10^4^ cells/well). The cells were pretreated with or without 10 μg/ml baicalin and 4 Gy X-ray dose. Protein lysates were separated by sodium dodecyl sulfate polyacrylamide gel electrophoresis and transferred to nitrocellulose membranes (Millipore, Billerica, MA). The membranes were blocked at room temperature for 1 h in 5% skim milk in Tris-buffered saline with Tween 20 and then incubated overnight at 4 °C with the following indicated primary antibodies: anti-LC3A/B (Abcam, Cambridge, UK), BECN1 polyclonal (ABclonal, US), and GAPDH antibodies (Abcam, Cambridge, UK). Goat anti-rabbit IgG was used as the secondary antibody (Abcam, Cambridge, UK). Fluorophores were detected using the Infrared Fluorescence Imaging System (BioRad Universal Hood III).

### Immunofluorescence assays

Cells were cultured on coverslips and subjected to treatments as indicated. Paraformaldehyde-fixed cells were stained with anti-LC3B antibody (Abcam Inc., Cambridge, MA, USA) and FITC antibody conjugate (Solarbio Inc., China, Beijing) secondary antibody and examined using a fluorescence microscope (Carl Zeiss LSM780, Instrument Development Center, NCKU).

### Transmission electron microscopy

Cells were trypsinized and harvested and then fixed for 1 h in a solution containing 2.5% glutaraldehyde and 2% paraformaldehyde in 0.1 M cacodylate buffer at pH 7.3. After fixation, the samples were post-fixed with buffer containing 1% OsO_4_ for 30 min. Ultra-thin sections were subsequently observed under a transmission electron microscope (JEOL JEM-1200EX, Japan) at 100 keV.

### Statistical analysis

Data are presented as the mean ± SD of at least three independent experiments. The results were tested for significance using the unpaired Student’s *t* test.

## Results

### Radioresistance of CNE-2R cell line in comparison with CNE-2 cell line

**Fig. 1:**
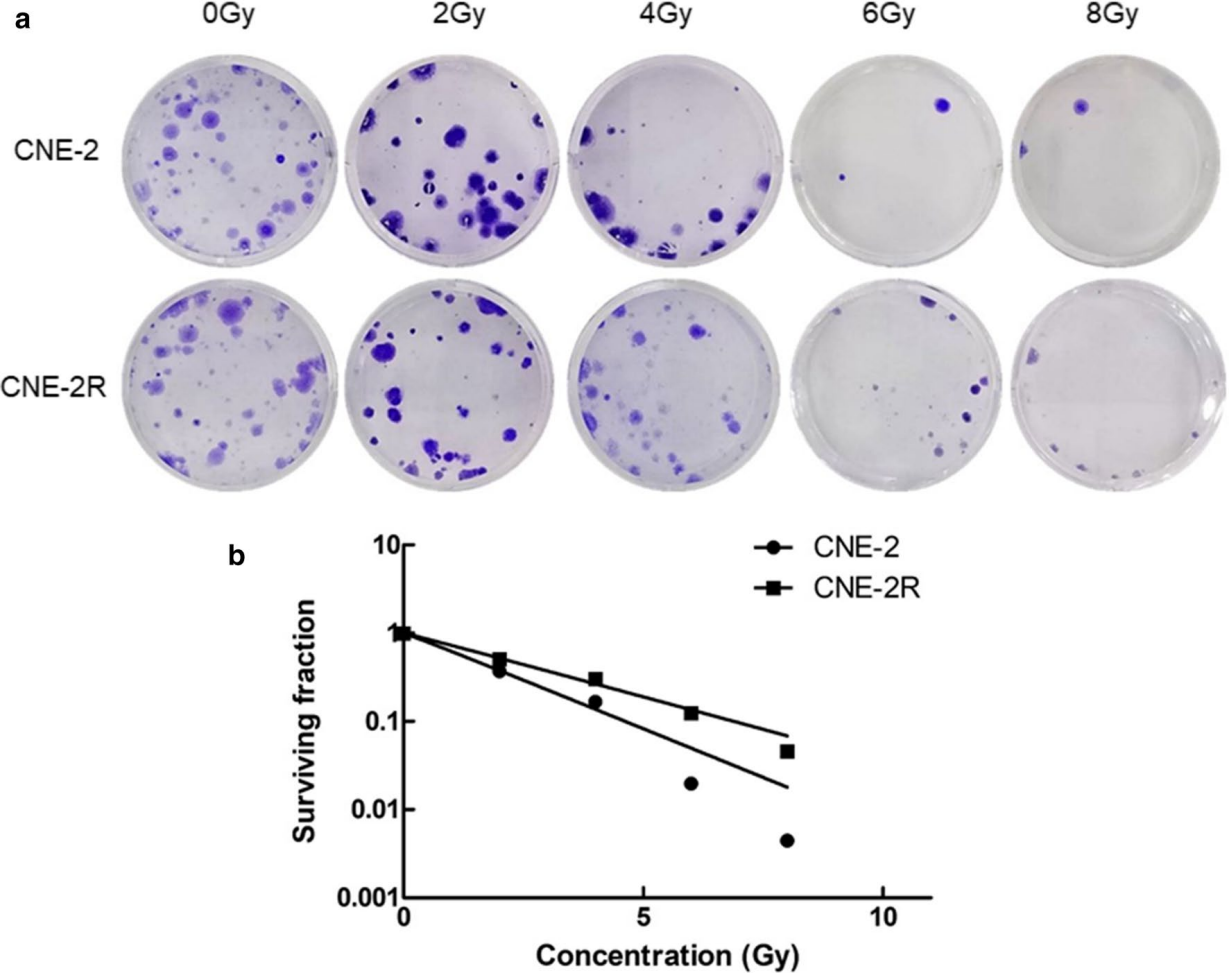
The radioresistance of CNE-2R cell lines confirmed by colony formation assays compared with the CNE-2 cell lines. **a** Colonies between the CNE-2 and CNE-2R cell lines detected by colony formation assays to testified the radioresistance of CNE-2R. **b** The curves were produced by multitarget model with the GraphPad Prism 5.0 software. The results were replicated in three independent tests

The radiosensitivity of the CNE-2 and CNE-2R cell lines was evaluated by colony formation assays after radiotherapy. As shown in Fig. 1a, no evident difference in colonies was observed between the two cell lines before radiotherapy. Nevertheless, noticeable differences in colonies were revealed at doses of 2 to 8 Gy after radiotherapy. Figure 1b shows the analysis of the cell survival curves of the two cell lines using the multi-target single-hit model, The analysis revealed significant differences in the main biological parameters between the two cell lines. Table 1 shows that survival fraction values of CNE-2R at 2 Gy (SF_2_) were 1.24 ± 0.029, and were evidently lower than SF_2_ of CNE-2 cell line (1.46 ± 0.013, P < 0.05). The repair capability from CNE-2R radiotherapy was higher than that of CNE-2. These findings suggest that CNE-2R was more radioresistant than that of CNE-2.

**Table 1:** Correspondence factors of the multitarget single-hit model

Cells	D_0_ (Gy)	D_q_ (Gy)	SF_2_
CNE-2R	1.96 ± 0.317	0.11 ± 0.025	1.24 ± 0.029
CNE-2	2.94 ± 0.231	0.32 ± 0.012	1.46 ± 0.013
P	< 0.05	< 0.05	< 0.05

D_0_ is the mean lethal dose; D_q_ is the required threshold for cell damage; SF_2_ is the survival fraction at 2 Gy

### Influence of baicalin in proliferation and apoptosis of CNE-2R cell line

**Fig. 2:**
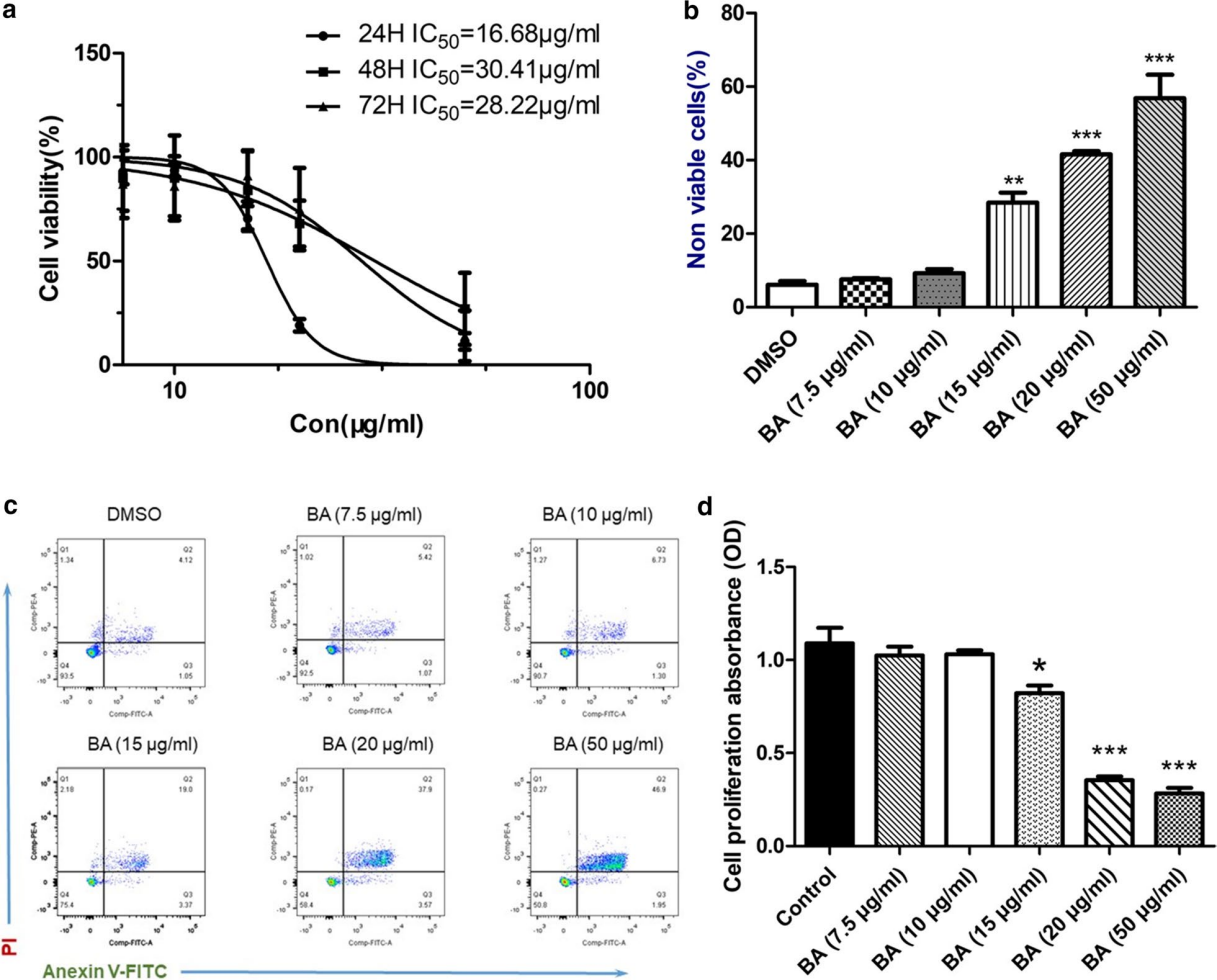
The cytotoxicity of baicalin with the CNE-2R cell lines. **a** The IC50 values of baicalin on the proliferation of CNE-2R cell lines in different drug influence times. **b**, **c** The influence of CNE-2R on apoptosis with different concentrations of baicalin. Apoptosis was identified by Annexin V/PI staining. Early apoptotic cells are revealed in the lower right quadrant of the chart, while the upper right quadrant showed late apoptotic cells (Annexin V +/PI +). The data of histogram was presented as the mean ± standard deviation of 3 replicates. **d** The impact of baicalin on the proliferation of CNE-2R in 24 h. Cell viability was determined by the CCK-8 assay and expressed with O.D. values. Data were presented as mean ± SD. *P < 0.05 and ***P < 0.001 were contrasted with the control group

The inhibitory effect of baicalin on CNE-2R cells was determined by the CCK-8 assay. The results showed that baicalin had a half-maximal inhibitory concentration (IC50) of 16.68, 30.41, and 28.22 μg/ml for CNE-2R cells at 24, 48, and 72 h, respectively (Fig. 2a). This result suggests that baicalin is a proliferative inhibitor of CNE-2R cell, and the maximum inhibitory time is 24 h. The proliferation of CNE-2R cells after 24 h of baicalin exposure in different concentrations was then investigated. The inhibition of proliferation was increased when the concentration rose to more than 20 μg/ml. No remarkable differences in inhibition were observed in the groups of DMSO and 7.5, 10, and 15 μg/ml (Fig. 2b).

Apoptosis was analyzed by flow cytometry to further investigate apoptotic potential of baicalin (Fig. 2c). Histogram data were presented as the mean ± standard deviation (Fig. 2d). The percentage of early and late stage apoptotic and necrotic cells had varying increment degrees in baicalin-treated groups compared with those in the control group. The percentages of total apoptosis were 6.51%, 9.3%, 24.55%, 41.64% and 49.12% in the control cells and those treated with 10 μg/ml (P > 0.05), 15 μg/ml (P < 0.01), 20 and 50 μg/ml of baicalin (P < 0.001). These results verified that baicalin induces CNE-2R cells apoptosis.

Therefore, this portion of the study aims to demonstrate the inhibition of cell viability and apoptotic effect of baicalin. A total of 10 μg/ml of baicalin was found to have lower inhibition and apoptotic effect than that of 20 μg/ml baicalin, and no considerable differences were found between the 10 μg/ml and control groups. Based on the experimental evidence, 10 μg/ml was selected as a reasonable dose for subsequent experiments.

### Baicalin reverses the radioresistance of CNE-2R cell

**Fig. 3:**
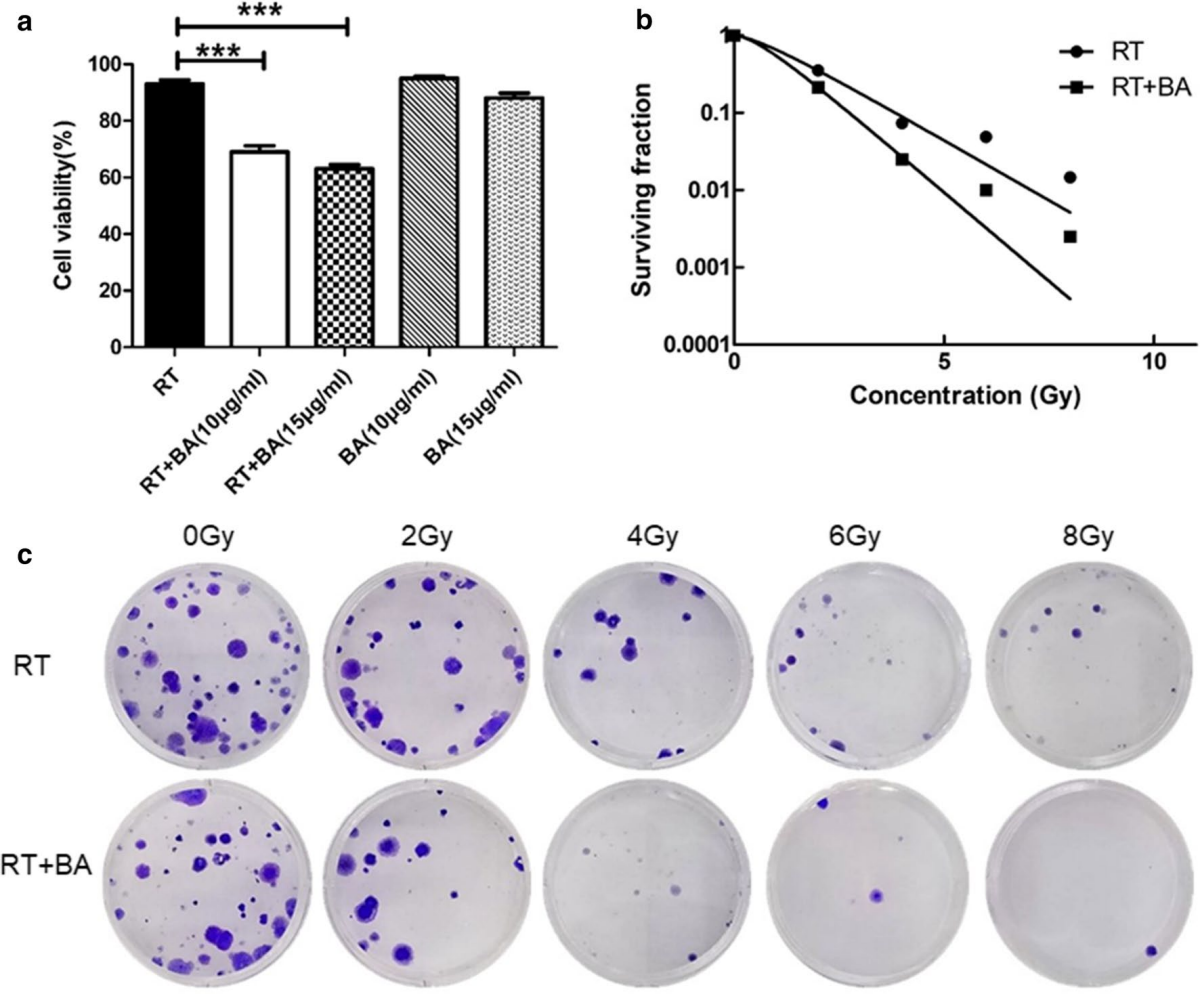
Baicalin reverse the radioresistance of CNE-2R cells. **a** Cell viability was determined by the CCK-8 assay and expressed with O.D. values. Data were presented as mean ± SD. ***P < 0.001 were contrasted with the radiotherapy group. **b** Colony formation assays detected to testified the radioresistance of the radiotherapy group and the baicalin group. **c** The curves were produced by multitarget model with the GraphPad Prism 5.0 software. The results were replicated in three independent tests

Colony-formation assays were conducted to evaluate the radiosensitivity between radiotherapy and radiotherapy combined baicalin (RT + BA) groups (Fig. 3a). Cell survival curves were fitted with the multitarget model in the GraphPad Prism 5.0 software (Fig. 3b). The survival rates of the RT + BA group were lower than that of the RT group at 2, 4, 6, and 8 Gy. The main biological parameters associated with radiotherapy based on the Dq and SF_2_ values are shown in Table 2. The survival fraction values of RT + BA group at 2 Gy (SF_2_) were 0.85 ± 0.029, and it was evidently lower than SF_2_ of RT group (1.19 ± 0.031, P < 0.05).

**Table 2:** Correspondence factors of the multitarget single-hit model

Treatment	D_0_ (Gy)	D_q_ (Gy)	SF_2_
RT	1.39 ± 0.201	0.33 ± 0.011	1.19 ± 0.031
RT + BA	0.944 ± 0.109	0.66 ± 0.032	0.85 ± 0.029
P	< 0.05	< 0.05	< 0.05

D_0_ is the mean lethal dose; D_q_ is the required threshold for cell damage; SF_2_ is the survival fraction at 2 Gy; RT (radiotherapy); BA (baicalin)

CCK-8 assays were used to evaluate the difference in proliferation inhibition after radiotherapy. The RT + BA group at 4 Gy radiation dose and 10 μg/ml concentration exhibited considerably higher cell proliferation inhibition than that of the RT and baicalin groups (Fig. 3c).

These results suggest that baicalin reverses the radioresistance and enhance radiosensitivity of CNE2R cells.

### Baicalin regulates the radioresistance of CNE-2R cells via autophagy

Based on the previous experiment, the radioresistance reversal of CNE-2R cells at 4 Gy radiation dose by baicalin and 10 μg/ml baicalin concentration was verified.

**Fig. 4:**
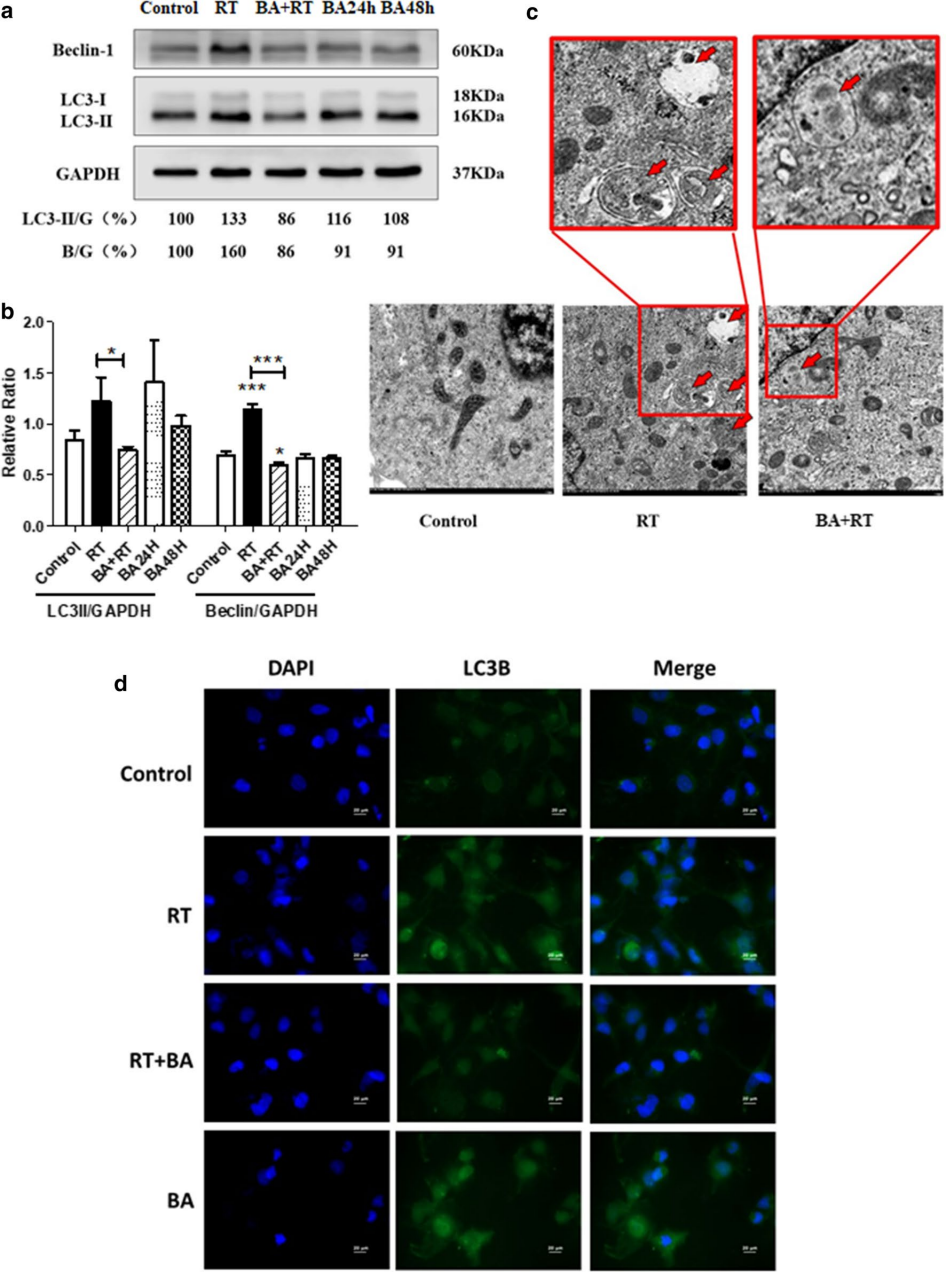
Effects of baicalin and radiotherapy on cells autophagy regulators by western blotting, immunofluorescence and electro-microscope. **a** Blots were analyzed with Beclin-1 and LC3I/II protein. **b** Error bars showed standard deviation from triplicates. A typical result from three independent experiments was presented, *P < 0.05. **c** Electron micrographs of CNE-2R cells exposed to baicalin, radiotherapy or radiotherapy combined with baicalin. Arrows indicate typical intracellular vesicles. **d** At 24 h after ionizing radiation of 4 Gy, LC3B fluorescence staining. Magnification ×40

In the initial analysis, the 4 Gy radiation dose and 10 μg/ml baicalin concentration treated with CNE-2R cells (RT + BA) were compared with the control, radiotherapy with 4 Gy radiation dose and 10 μg/ml baicalin concentration groups. Western blot analysis experiments were conducted to examine the expression levels of autophagy-associated proteins, namely, LC3 I/II and Beclin-1, in the four groups 24 h after radiotherapy. The LC3-II levels increased after radiotherapy (Fig. 4a). Nevertheless, when cells were pretreated with baicalin 24 h before radiotherapy, the increase in LC3-II expression was abrogated (P < 0.05; Fig. 4b). LC3 II/GAPDH ratio in the combined group (RT + BA) was notably lower than that in the radiotherapy group (RT).

Figure 4c shows the autophagosome formation of CNE-2R. Electron microscopy results demonstrated that the emergence of autophagy vacuoles began in the radiotherapy group at dose of 4 Gy after 24 h. Additional autophagy vacuoles in the radiotherapy group were found. However, the autophagy vacuoles were reduced when the cells were pretreated with 10 μg/ml baicalin 24 h before irradiation.

The aforementioned results demonstrate the upregulated function of radiotherapy in autophagy. Baicalin can also downregulate the abnormally elevated autophagy by radiotherapy without reducing the non-radiotherapy CNE-2R cells.

LC3B expression level correlates with the extent of autophagosome formation. It can be used as an autophagosomal marker. LC3B staining was detected in CNE-2R to reconfirm the effects of baicalin in reversed radioresistance (Fig. 4d). Immunofluorescence results showed that radiation increased LC3B expression in contrast with the control group. Moreover, results show that pretreatment with 10 μg/ml baicalin 24 h before irradiation with 4 Gy X-ray decreased LC3B generation compared with the radiotherapy group. However, the expression of non-radiotherapy cells was not affected by baicalin.

## Discussion

Numerous studies have confirmed that radiotherapy resistance is closely related to recurrence after radiotherapy for nasopharyngeal carcinoma. Therefore, the human nasopharyngeal carcinoma radiotherapy-resistant cell line CNE-2R was used as the research object. The clone formation study was also utilized to compare differences in the radiotherapy sensitive cell line CNE-2 to confirm the radioresistance of CNE-2R cell line at the beginning of the study. As shown in Fig. 2a, no evident difference in colonies was found between these two cell lines before radiotherapy, but evident differences in colonies were revealed at doses of 2 to 8 Gy after radiotherapy. Evidence suggests that CNE-2R cells were radioresistant.

Radiotherapy can induce cancer cell autophagy through multiple pathways. This type of treatment can directly or indirectly damage DNA and activate DNA damage repair signaling pathways, such as p53, ATM, PARP, FOXO3a, and mTOR [5]. Radiation can also damage nuclear extracellular targets, such as cell membrane, mitochondria, and endoplasmic reticulum. This damage can lead to ceramide accumulation, Ca^2+^ concentration, ROS increase, activating various stress signaling pathways to regulate autophagy. Among these condition, ceramide can cause ER stress and mitochondrial dysfunction, thereby triggering autophagy. ROS, an important cytoplasmic signaling pathway activator, including p38, JNK, and HIF-1α, activates autophagy-related signaling pathways. ROS can also cause damage to mitochondria and ER, thereby increasing ROS and Ca^2+^ levels, which are membrane sensors that activate endoplasmic reticulum stress and trigger autophagy [10]. Autophagy levels are inversely related to the radiosensitivity of human nasopharyngeal carcinoma cell lines. The sensitivity of CNE-2R to radiation therapy increased with autophagy inhibition [6, 11]. The same results were also observed in this study. The LC3-II level detected in the radiotherapy group was considerably higher than that in the control group after 4 Gy radiation dose and 24 h after RT. Consequently, radiation therapy can increase autophagy in CNE-2R cell line.

Baicalin, which is derived from *S. baicalensis*, is an important constituent of traditional Chinese medicine. Baicalin reduces growth of nasopharyngeal carcinoma in vivo and in vitro, and induces cell cycle arrest and apoptosis [9]. Baicalin regulates autophagy in different cancer cells [12]. However, any notable autophagy inhibition by baicalin acting alone in the CNE-2R cell line was not observed. Baicalin alone with CNE-2R cells can still inhibit cell growth and promote apoptosis. Immunofluorescence and Western blot analysis confirmed that baicalin could substantially downregulate elevated LC3 levels of radiotherapy pretreated with baicalin before radiotherapy. The results of electron microscopy also showed that numerous autophagic vacuoles appeared in the tumor cells after radiotherapy, and the application of baicalin could reduce the autophagic vacuoles. Autophagy vacuole is a typical morphological change of autophagy, which can confirm the occurrence of autophagy. Baicalin reduces the increase in radiation therapy-induced autophagy from ultrastructure. The enhancement of autophagy, inhibition of tumor growth via baicalin alone, and the reduction of autophagy combined radiotherapy remain unknown. Furthermore, autophagy-related signaling pathways affected by baicalin will be determined.

## Conclusion

Overall, baicalin can reverse the radioresistance of human nasopharyngeal carcinoma CNE-2R cells by downregulating radiotherapy-enhanced autophagy. Baicalin is the index component of *S. baicalensis* and used in the treatment of hepatitis for a long time. Our findings may extend the indications of drugs to a broader range.

## Data Availability

Not applicable.
